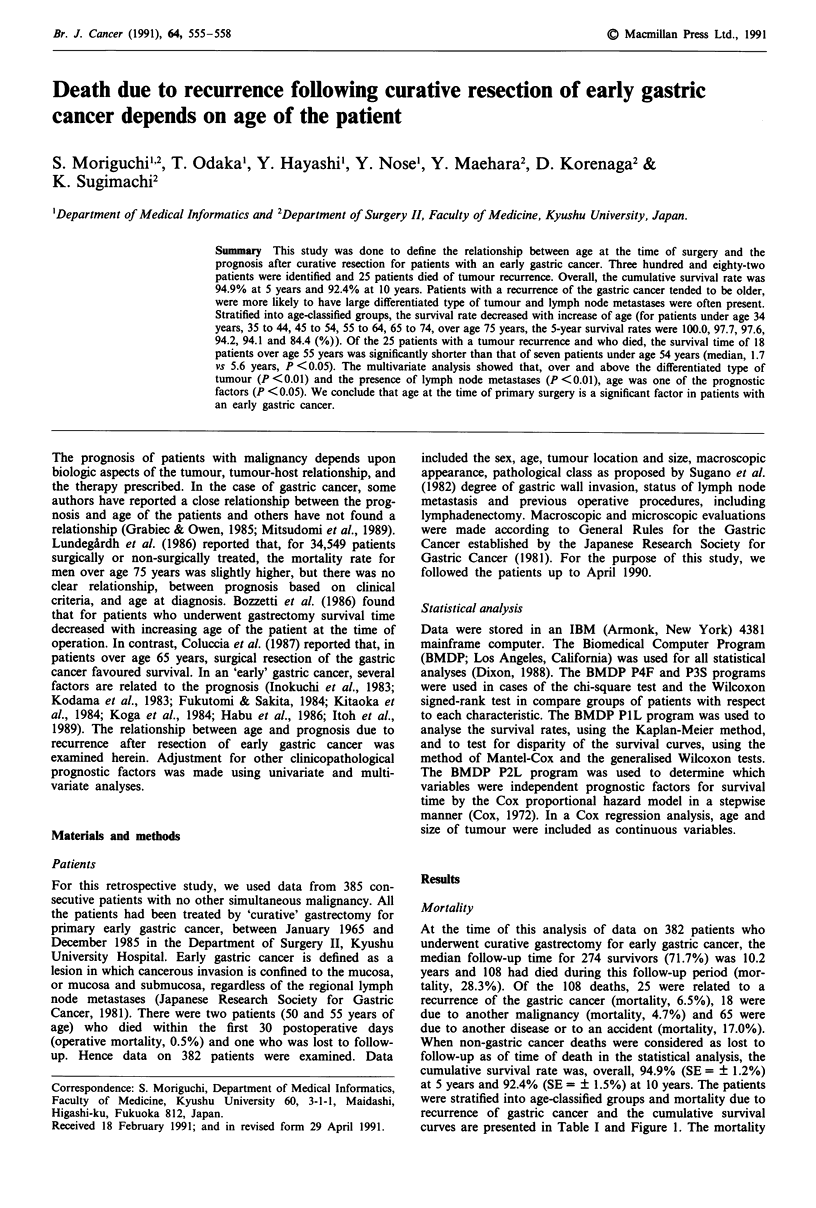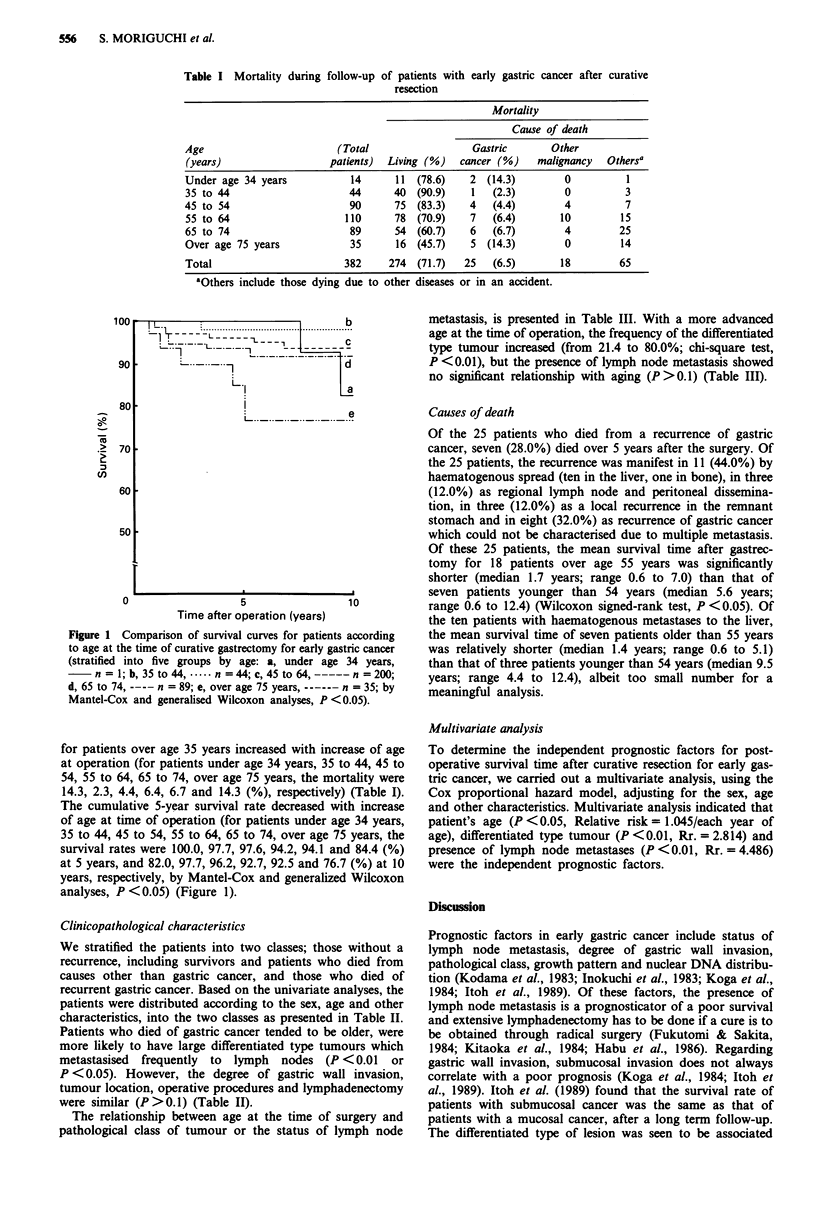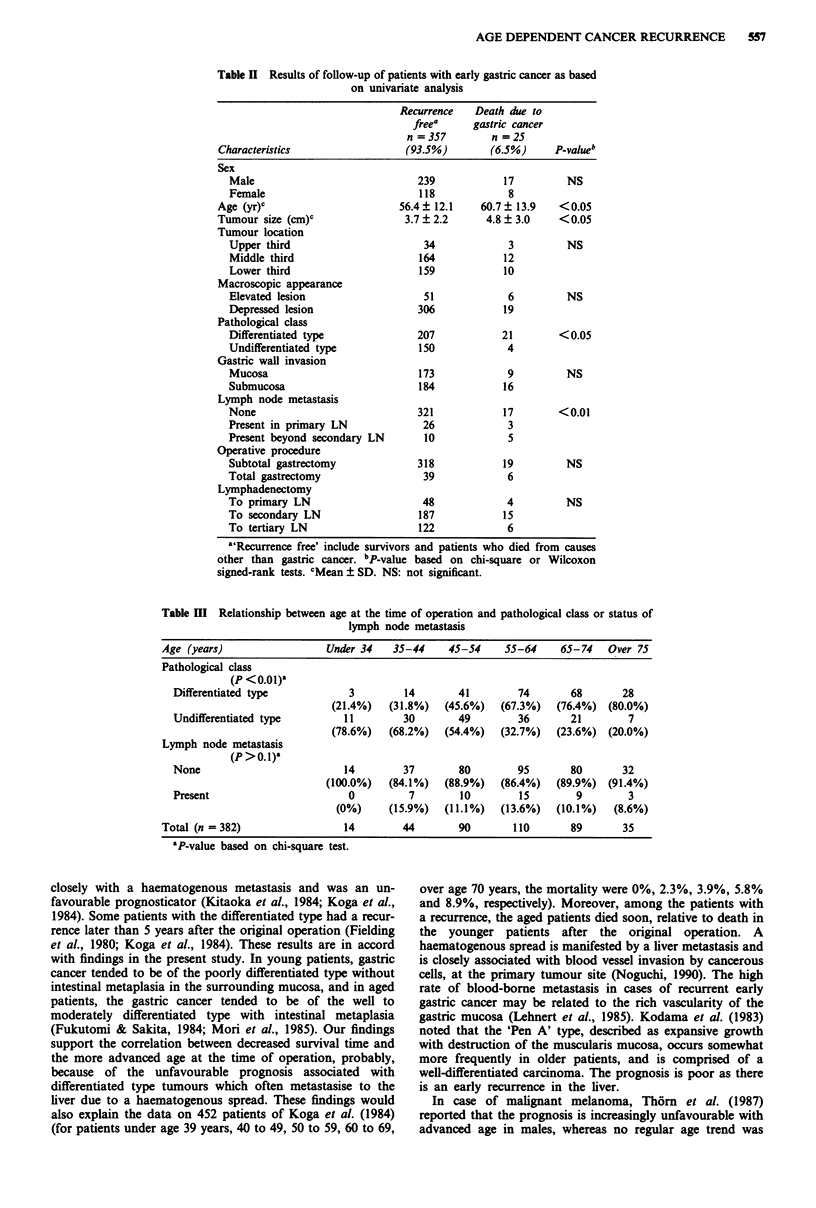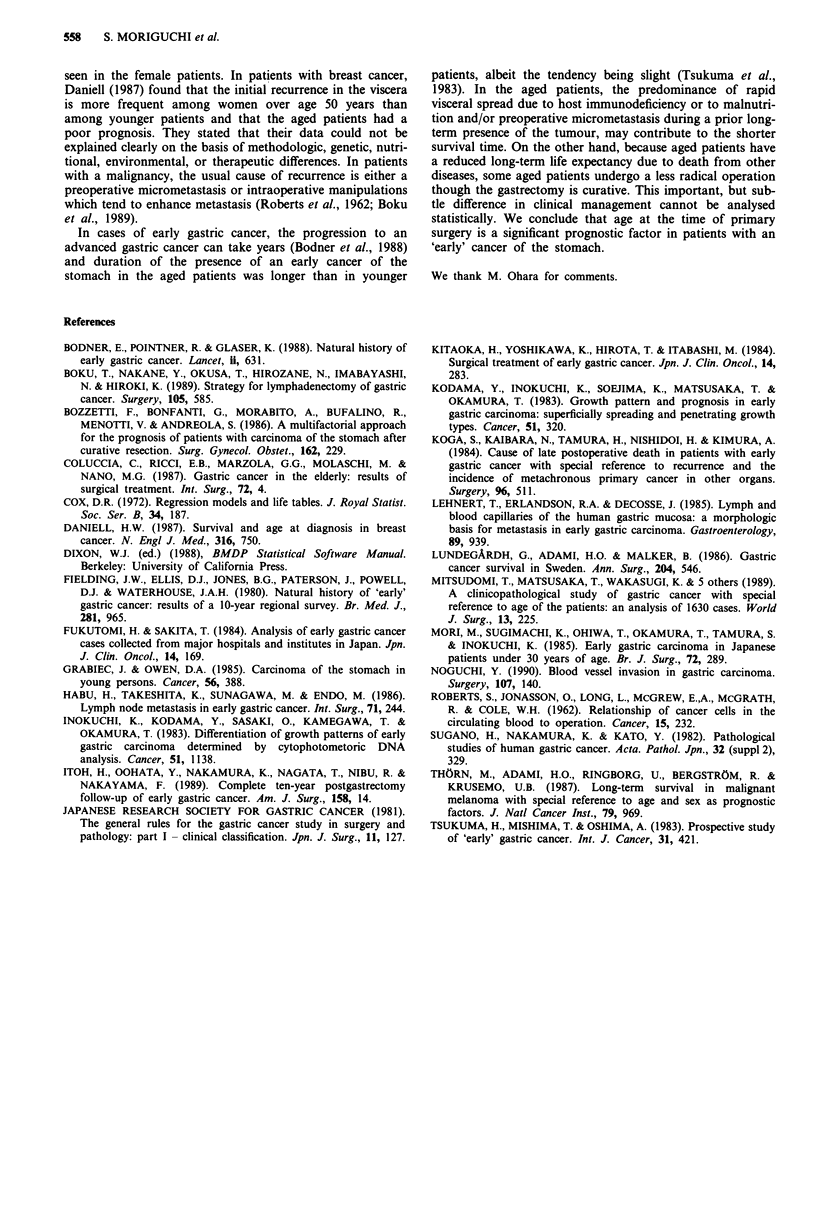# Death due to recurrence following curative resection of early gastric cancer depends on age of the patient.

**DOI:** 10.1038/bjc.1991.349

**Published:** 1991-09

**Authors:** S. Moriguchi, T. Odaka, Y. Hayashi, Y. Nose, Y. Maehara, D. Korenaga, K. Sugimachi

**Affiliations:** Department of Medical Informatics, Faculty of Medicine, Kyushu University, Japan.

## Abstract

This study was done to define the relationship between age at the time of surgery and the prognosis after curative resection for patients with an early gastric cancer. Three hundred and eighty-two patients were identified and 25 patients died of tumour recurrence. Overall, the cumulative survival rate was 94.9% at 5 years and 92.4% at 10 years. Patients with a recurrence of the gastric cancer tended to be older, were more likely to have large differentiated type of tumour and lymph node metastases were often present. Stratified into age-classified groups, the survival rate decreased with increase of age (for patients under age 34 years, 35 to 44, 45 to 54, 55 to 64, 65 to 74, over age 75 years, the 5-year survival rates were 100.0, 97.7, 97.6, 94.2, 94.1 and 84.4 (%]. Of the 25 patients with a tumour recurrence and who died, the survival time of 18 patients over age 55 years was significantly shorter than that of seven patients under age 54 years (median, 1.7 vs 5.6 years, P less than 0.05). The multivariate analysis showed that, over and above the differentiated type of tumour (P less than 0.01) and the presence of lymph node metastases (P less than 0.01), age was one of the prognostic factors (P less than 0.05). We conclude that age at the time of primary surgery is a significant factor in patients with an early gastric cancer.


*Br. J. Cancer* (1991), **64**, 555–558 © Macmillan Press Ltd., 1991

### Death due to recurrence following curative resection of early gastric cancer depends on age of the patient

S. Moriguchi[1,2], T. Odaka[1], Y. Hayashi[1], Y. Nose[1], Y. Maehara[2], D. Korenaga[2] & K. Sugimachi[2]

[1]*Department of Medical Informatics and* [2]*Department of Surgery II, Faculty of Medicine, Kyushu University, Japan.*

**Summary** This study was done to define the relationship between age at the time of surgery and the prognosis after curative resection for patients with an early gastric cancer. Three hundred and eighty-two patients were identified and 25 patients died of tumour recurrence. Overall, the cumulative survival rate was 94.9% at 5 years and 92.4% at 10 years. Patients with a recurrence of the gastric cancer tended to be older, were more likely to have large differentiated type of tumour and lymph node metastases were often present. Stratified into age-classified groups, the survival rate decreased with increase of age (for patients under age 34 years, 35 to 44, 45 to 54, 55 to 64, 65 to 74, over age 75 years, the 5-year survival rates were 100.0, 97.7, 97.6, 94.2, 94.1 and 84.4 (%)). Of the 25 patients with a tumour recurrence and who died, the survival time of 18 patients over age 55 years was significantly shorter than that of seven patients under age 54 years (median, 1.7 *vs* 5.6 years, $P < 0.05$). The multivariate analysis showed that, over and above the differentiated type of tumour ($P < 0.01$) and the presence of lymph node metastases ($P < 0.01$), age was one of the prognostic factors ($P < 0.05$). We conclude that age at the time of primary surgery is a significant factor in patients with an early gastric cancer.

The prognosis of patients with malignancy depends upon biologic aspects of the tumour, tumour-host relationship, and the therapy prescribed. In the case of gastric cancer, some authors have reported a close relationship between the prognosis and age of the patients and others have not found a relationship (Grabiec & Owen, 1985; Mitsudomi *et al.*, 1989). Lundegårdh *et al.* (1986) reported that, for 34,549 patients surgically or non-surgically treated, the mortality rate for men over age 75 years was slightly higher, but there was no clear relationship, between prognosis based on clinical criteria, and age at diagnosis. Bozzetti *et al.* (1986) found that for patients who underwent gastrectomy survival time decreased with increasing age of the patient at the time of operation. In contrast, Coluccia *et al.* (1987) reported that, in patients over age 65 years, surgical resection of the gastric cancer favoured survival. In an 'early' gastric cancer, several factors are related to the prognosis (Inokuchi *et al.*, 1983; Kodama *et al.*, 1983; Fukutomi & Sakita, 1984; Kitaoka *et al.*, 1984; Koga *et al.*, 1984; Habu *et al.*, 1986; Itoh *et al.*, 1989). The relationship between age and prognosis due to recurrence after resection of early gastric cancer was examined herein. Adjustment for other clinicopathological prognostic factors was made using univariate and multivariate analyses.

### Materials and methods

### *Patients*

For this retrospective study, we used data from 385 consecutive patients with no other simultaneous malignancy. All the patients had been treated by 'curative' gastrectomy for primary early gastric cancer, between January 1965 and December 1985 in the Department of Surgery II, Kyushu University Hospital. Early gastric cancer is defined as a lesion in which cancerous invasion is confined to the mucosa, or mucosa and submucosa, regardless of the regional lymph node metastases (Japanese Research Society for Gastric Cancer, 1981). There were two patients (50 and 55 years of age) who died within the first 30 postoperative days (operative mortality, 0.5%) and one who was lost to follow-up. Hence data on 382 patients were examined. Data included the sex, age, tumour location and size, macroscopic appearance, pathological class as proposed by Sugano *et al.* (1982) degree of gastric wall invasion, status of lymph node metastasis and previous operative procedures, including lymphadenectomy. Macroscopic and microscopic evaluations were made according to General Rules for the Gastric Cancer established by the Japanese Research Society for Gastric Cancer (1981). For the purpose of this study, we followed the patients up to April 1990.

### *Statistical analysis*

Data were stored in an IBM (Armonk, New York) 4381 mainframe computer. The Biomedical Computer Program (BMDP; Los Angeles, California) was used for all statistical analyses (Dixon, 1988). The BMDP P4F and P3S programs were used in cases of the chi-square test and the Wilcoxon signed-rank test in compare groups of patients with respect to each characteristic. The BMDP P1L program was used to analyse the survival rates, using the Kaplan-Meier method, and to test for disparity of the survival curves, using the method of Mantel-Cox and the generalised Wilcoxon tests. The BMDP P2L program was used to determine which variables were independent prognostic factors for survival time by the Cox proportional hazard model in a stepwise manner (Cox, 1972). In a Cox regression analysis, age and size of tumour were included as continuous variables.

### Results

### *Mortality*

At the time of this analysis of data on 382 patients who underwent curative gastrectomy for early gastric cancer, the median follow-up time for 274 survivors (71.7%) was 10.2 years and 108 had died during this follow-up period (mortality, 28.3%). Of the 108 deaths, 25 were related to a recurrence of the gastric cancer (mortality, 6.5%), 18 were due to another malignancy (mortality, 4.7%) and 65 were due to another disease or to an accident (mortality, 17.0%). When non-gastric cancer deaths were considered as lost to follow-up as of time of death in the statistical analysis, the cumulative survival rate was, overall, 94.9% (SE = ± 1.2%) at 5 years and 92.4% (SE = ± 1.5%) at 10 years. The patients were stratified into age-classified groups and mortality due to recurrence of gastric cancer and the cumulative survival curves are presented in Table I and Figure 1. The mortality

Correspondence: S. Moriguchi, Department of Medical Informatics, Faculty of Medicine, Kyushu University 60, 3-1-1, Maidashi, Higashi-ku, Fukuoka 812, Japan.
Received 18 February 1991; and in revised form 29 April 1991.

**Table I** Mortality during follow-up of patients with early gastric cancer after curative resection

| | | Mortality | | | |
|---|---|---|---|---|---|
| | | | Cause of death | | |
| *Age (years)* | *(Total patients)* | *Living (%)* | *Gastric cancer (%)* | *Other malignancy* | *Others*[a] |
| Under age 34 years | 14 | 11 (78.6) | 2 (14.3) | 0 | 1 |
| 35 to 44 | 44 | 40 (90.9) | 1 (2.3) | 0 | 3 |
| 45 to 54 | 90 | 75 (83.3) | 4 (4.4) | 4 | 7 |
| 55 to 64 | 110 | 78 (70.9) | 7 (6.4) | 10 | 15 |
| 65 to 74 | 89 | 54 (60.7) | 6 (6.7) | 4 | 25 |
| Over age 75 years | 35 | 16 (45.7) | 5 (14.3) | 0 | 14 |
| Total | 382 | 274 (71.7) | 25 (6.5) | 18 | 65 |

[a]Others include those dying due to other diseases or in an accident.

**Figure 1** Comparison of survival curves for patients according to age at the time of curative gastrectomy for early gastric cancer (stratified into five groups by age: **a**, under age 34 years, —— $n = 1$; **b**, 35 to 44, ····· $n = 44$; **c**, 45 to 64, ------ $n = 200$; **d**, 65 to 74, ---- $n = 89$; **e**, over age 75 years, ------ $n = 35$; by Mantel-Cox and generalised Wilcoxon analyses, $P < 0.05$).

for patients over age 35 years increased with increase of age at operation (for patients under age 34 years, 35 to 44, 45 to 54, 55 to 64, 65 to 74, over age 75 years, the mortality were 14.3, 2.3, 4.4, 6.4, 6.7 and 14.3 (%), respectively) (Table I). The cumulative 5-year survival rate decreased with increase of age at time of operation (for patients under age 34 years, 35 to 44, 45 to 54, 55 to 64, 65 to 74, over age 75 years, the survival rates were 100.0, 97.7, 97.6, 94.2, 94.1 and 84.4 (%) at 5 years, and 82.0, 97.7, 96.2, 92.7, 92.5 and 76.7 (%) at 10 years, respectively, by Mantel-Cox and generalized Wilcoxon analyses, $P < 0.05$) (Figure 1).

### *Clinicopathological characteristics*

We stratified the patients into two classes; those without a recurrence, including survivors and patients who died from causes other than gastric cancer, and those who died of recurrent gastric cancer. Based on the univariate analyses, the patients were distributed according to the sex, age and other characteristics, into the two classes as presented in Table II. Patients who died of gastric cancer tended to be older, were more likely to have large differentiated type tumours which metastasised frequently to lymph nodes ($P < 0.01$ or $P < 0.05$). However, the degree of gastric wall invasion, tumour location, operative procedures and lymphadenectomy were similar ($P > 0.1$) (Table II).

The relationship between age at the time of surgery and pathological class of tumour or the status of lymph node metastasis, is presented in Table III. With a more advanced age at the time of operation, the frequency of the differentiated type tumour increased (from 21.4 to 80.0%; chi-square test, $P < 0.01$), but the presence of lymph node metastasis showed no significant relationship with aging ($P > 0.1$) (Table III).

### *Causes of death*

Of the 25 patients who died from a recurrence of gastric cancer, seven (28.0%) died over 5 years after the surgery. Of the 25 patients, the recurrence was manifest in 11 (44.0%) by haematogenous spread (ten in the liver, one in bone), in three (12.0%) as regional lymph node and peritoneal dissemination, in three (12.0%) as a local recurrence in the remnant stomach and in eight (32.0%) as recurrence of gastric cancer which could not be characterised due to multiple metastasis. Of these 25 patients, the mean survival time after gastrectomy for 18 patients over age 55 years was significantly shorter (median 1.7 years; range 0.6 to 7.0) than that of seven patients younger than 54 years (median 5.6 years; range 0.6 to 12.4) (Wilcoxon signed-rank test, $P < 0.05$). Of the ten patients with haematogenous metastases to the liver, the mean survival time of seven patients older than 55 years was relatively shorter (median 1.4 years; range 0.6 to 5.1) than that of three patients younger than 54 years (median 9.5 years; range 4.4 to 12.4), albeit too small number for a meaningful analysis.

### *Multivariate analysis*

To determine the independent prognostic factors for post-operative survival time after curative resection for early gastric cancer, we carried out a multivariate analysis, using the Cox proportional hazard model, adjusting for the sex, age and other characteristics. Multivariate analysis indicated that patient's age ($P < 0.05$, Relative risk = 1.045/each year of age), differentiated type tumour ($P < 0.01$, Rr. = 2.814) and presence of lymph node metastases ($P < 0.01$, Rr. = 4.486) were the independent prognostic factors.

### Discussion

Prognostic factors in early gastric cancer include status of lymph node metastasis, degree of gastric wall invasion, pathological class, growth pattern and nuclear DNA distribution (Kodama *et al.*, 1983; Inokuchi *et al.*, 1983; Koga *et al.*, 1984; Itoh *et al.*, 1989). Of these factors, the presence of lymph node metastasis is a prognosticator of a poor survival and extensive lymphadenectomy has to be done if a cure is to be obtained through radical surgery (Fukutomi & Sakita, 1984; Kitaoka *et al.*, 1984; Habu *et al.*, 1986). Regarding gastric wall invasion, submucosal invasion does not always correlate with a poor prognosis (Koga *et al.*, 1984; Itoh *et al.*, 1989). Itoh *et al.* (1989) found that the survival rate of patients with submucosal cancer was the same as that of patients with a mucosal cancer, after a long term follow-up. The differentiated type of lesion was seen to be associated

**Table II** Results of follow-up of patients with early gastric cancer as based on univariate analysis

| *Characteristics* | *Recurrence free[a]* *n = 357* *(93.5%)* | *Death due to gastric cancer* *n = 25* *(6.5%)* | *P-value[b]* |
|---|---|---|---|
| Sex | | | |
| Male | 239 | 17 | NS |
| Female | 118 | 8 | |
| Age (yr)[c] | 56.4 ± 12.1 | 60.7 ± 13.9 | <0.05 |
| Tumour size (cm)[c] | 3.7 ± 2.2 | 4.8 ± 3.0 | <0.05 |
| Tumour location | | | |
| Upper third | 34 | 3 | NS |
| Middle third | 164 | 12 | |
| Lower third | 159 | 10 | |
| Macroscopic appearance | | | |
| Elevated lesion | 51 | 6 | NS |
| Depressed lesion | 306 | 19 | |
| Pathological class | | | |
| Differentiated type | 207 | 21 | <0.05 |
| Undifferentiated type | 150 | 4 | |
| Gastric wall invasion | | | |
| Mucosa | 173 | 9 | NS |
| Submucosa | 184 | 16 | |
| Lymph node metastasis | | | |
| None | 321 | 17 | <0.01 |
| Present in primary LN | 26 | 3 | |
| Present beyond secondary LN | 10 | 5 | |
| Operative procedure | | | |
| Subtotal gastrectomy | 318 | 19 | NS |
| Total gastrectomy | 39 | 6 | |
| Lymphadenectomy | | | |
| To primary LN | 48 | 4 | NS |
| To secondary LN | 187 | 15 | |
| To tertiary LN | 122 | 6 | |

[a]'Recurrence free' include survivors and patients who died from causes other than gastric cancer. [b]*P*-value based on chi-square or Wilcoxon signed-rank tests. [c]Mean ± SD. NS: not significant.

**Table III** Relationship between age at the time of operation and pathological class or status of lymph node metastasis

| *Age (years)* | *Under 34* | *35–44* | *45–54* | *55–64* | *65–74* | *Over 75* |
|---|---|---|---|---|---|---|
| Pathological class ($P < 0.01$)[a] | | | | | | |
| Differentiated type | 3 (21.4%) | 14 (31.8%) | 41 (45.6%) | 74 (67.3%) | 68 (76.4%) | 28 (80.0%) |
| Undifferentiated type | 11 (78.6%) | 30 (68.2%) | 49 (54.4%) | 36 (32.7%) | 21 (23.6%) | 7 (20.0%) |
| Lymph node metastasis ($P > 0.1$)[a] | | | | | | |
| None | 14 (100.0%) | 37 (84.1%) | 80 (88.9%) | 95 (86.4%) | 80 (89.9%) | 32 (91.4%) |
| Present | 0 (0%) | 7 (15.9%) | 10 (11.1%) | 15 (13.6%) | 9 (10.1%) | 3 (8.6%) |
| Total ($n$ = 382) | 14 | 44 | 90 | 110 | 89 | 35 |

[a]*P*-value based on chi-square test.

closely with a haematogenous metastasis and was an unfavourable prognosticator (Kitaoka *et al.*, 1984; Koga *et al.*, 1984). Some patients with the differentiated type had a recurrence later than 5 years after the original operation (Fielding *et al.*, 1980; Koga *et al.*, 1984). These results are in accord with findings in the present study. In young patients, gastric cancer tended to be of the poorly differentiated type without intestinal metaplasia in the surrounding mucosa, and in aged patients, the gastric cancer tended to be of the well to moderately differentiated type with intestinal metaplasia (Fukutomi & Sakita, 1984; Mori *et al.*, 1985). Our findings support the correlation between decreased survival time and the more advanced age at the time of operation, probably, because of the unfavourable prognosis associated with differentiated type tumours which often metastasise to the liver due to a haematogenous spread. These findings would also explain the data on 452 patients of Koga *et al.* (1984) (for patients under age 39 years, 40 to 49, 50 to 59, 60 to 69, over age 70 years, the mortality were 0%, 2.3%, 3.9%, 5.8% and 8.9%, respectively). Moreover, among the patients with a recurrence, the aged patients died soon, relative to death in the younger patients after the original operation. A haematogenous spread is manifested by a liver metastasis and is closely associated with blood vessel invasion by cancerous cells, at the primary tumour site (Noguchi, 1990). The high rate of blood-borne metastasis in cases of recurrent early gastric cancer may be related to the rich vascularity of the gastric mucosa (Lehnert *et al.*, 1985). Kodama *et al.* (1983) noted that the 'Pen A' type, described as expansive growth with destruction of the muscularis mucosa, occurs somewhat more frequently in older patients, and is comprised of a well-differentiated carcinoma. The prognosis is poor as there is an early recurrence in the liver.

In case of malignant melanoma, Thörn *et al.* (1987) reported that the prognosis is increasingly unfavourable with advanced age in males, whereas no regular age trend was seen in the female patients. In patients with breast cancer, Daniell (1987) found that the initial recurrence in the viscera is more frequent among women over age 50 years than among younger patients and that the aged patients had a poor prognosis. They stated that their data could not be explained clearly on the basis of methodologic, genetic, nutritional, environmental, or therapeutic differences. In patients with a malignancy, the usual cause of recurrence is either a preoperative micrometastasis or intraoperative manipulations which tend to enhance metastasis (Roberts *et al.*, 1962; Boku *et al.*, 1989).

In cases of early gastric cancer, the progression to an advanced gastric cancer can take years (Bodner *et al.*, 1988) and duration of the presence of an early cancer of the stomach in the aged patients was longer than in younger patients, albeit the tendency being slight (Tsukuma *et al.*, 1983). In the aged patients, the predominance of rapid visceral spread due to host immunodeficiency or to malnutrition and/or preoperative micrometastasis during a prior long-term presence of the tumour, may contribute to the shorter survival time. On the other hand, because aged patients have a reduced long-term life expectancy due to death from other diseases, some aged patients undergo a less radical operation though the gastrectomy is curative. This important, but subtle difference in clinical management cannot be analysed statistically. We conclude that age at the time of primary surgery is a significant prognostic factor in patients with an 'early' cancer of the stomach.

We thank M. Ohara for comments.

### References

BODNER, E., POINTNER, R. & GLASER, K. (1988). Natural history of early gastric cancer. *Lancet*, **ii**, 631.

BOKU, T., NAKANE, Y., OKUSA, T., HIROZANE, N., IMABAYASHI, N. & HIROKI, K. (1989). Strategy for lymphadenectomy of gastric cancer. *Surgery*, **105**, 585.

BOZZETTI, F., BONFANTI, G., MORABITO, A., BUFALINO, R., MENOTTI, V. & ANDREOLA, S. (1986). A multifactorial approach for the prognosis of patients with carcinoma of the stomach after curative resection. *Surg. Gynecol. Obstet.*, **162**, 229.

COLUCCIA, C., RICCI, E.B., MARZOLA, G.G., MOLASCHI, M. & NANO, M.G. (1987). Gastric cancer in the elderly: results of surgical treatment. *Int. Surg.*, **72**, 4.

COX, D.R. (1972). Regression models and life tables. *J. Royal Statist. Soc. Ser. B*, **34**, 187.

DANIELL, H.W. (1987). Survival and age at diagnosis in breast cancer. *N. Engl J. Med.*, **316**, 750.

DIXON, W.J. (ed.) (1988), *BMDP Statistical Software Manual*. Berkeley: University of California Press.

FIELDING, J.W., ELLIS, D.J., JONES, B.G., PATERSON, J., POWELL, D.J. & WATERHOUSE, J.A.H. (1980). Natural history of 'early' gastric cancer: results of a 10-year regional survey. *Br. Med. J.*, **281**, 965.

FUKUTOMI, H. & SAKITA, T. (1984). Analysis of early gastric cancer cases collected from major hospitals and institutes in Japan. *Jpn. J. Clin. Oncol.*, **14**, 169.

GRABIEC, J. & OWEN, D.A. (1985). Carcinoma of the stomach in young persons. *Cancer*, **56**, 388.

HABU, H., TAKESHITA, K., SUNAGAWA, M. & ENDO, M. (1986). Lymph node metastasis in early gastric cancer. *Int. Surg.*, **71**, 244.

INOKUCHI, K., KODAMA, Y., SASAKI, O., KAMEGAWA, T. & OKAMURA, T. (1983). Differentiation of growth patterns of early gastric carcinoma determined by cytophotometoric DNA analysis. *Cancer*, **51**, 1138.

ITOH, H., OOHATA, Y., NAKAMURA, K., NAGATA, T., NIBU, R. & NAKAYAMA, F. (1989). Complete ten-year postgastrectomy follow-up of early gastric cancer. *Am. J. Surg.*, **158**, 14.

JAPANESE RESEARCH SOCIETY FOR GASTRIC CANCER (1981). The general rules for the gastric cancer study in surgery and pathology: part I – clinical classification. *Jpn. J. Surg.*, **11**, 127.

KITAOKA, H., YOSHIKAWA, K., HIROTA, T. & ITABASHI, M. (1984). Surgical treatment of early gastric cancer. *Jpn. J. Clin. Oncol.*, **14**, 283.

KODAMA, Y., INOKUCHI, K., SOEJIMA, K., MATSUSAKA, T. & OKAMURA, T. (1983). Growth pattern and prognosis in early gastric carcinoma: superficially spreading and penetrating growth types. *Cancer*, **51**, 320.

KOGA, S., KAIBARA, N., TAMURA, H., NISHIDOI, H. & KIMURA, A. (1984). Cause of late postoperative death in patients with early gastric cancer with special reference to recurrence and the incidence of metachronous primary cancer in other organs. *Surgery*, **96**, 511.

LEHNERT, T., ERLANDSON, R.A. & DECOSSE, J. (1985). Lymph and blood capillaries of the human gastric mucosa: a morphologic basis for metastasis in early gastric carcinoma. *Gastroenterology*, **89**, 939.

LUNDEGÅRDH, G., ADAMI, H.O. & MALKER, B. (1986). Gastric cancer survival in Sweden. *Ann. Surg.*, **204**, 546.

MITSUDOMI, T., MATSUSAKA, T., WAKASUGI, K. & 5 others (1989). A clinicopathological study of gastric cancer with special reference to age of the patients: an analysis of 1630 cases. *World J. Surg.*, **13**, 225.

MORI, M., SUGIMACHI, K., OHIWA, T., OKAMURA, T., TAMURA, S. & INOKUCHI, K. (1985). Early gastric carcinoma in Japanese patients under 30 years of age. *Br. J. Surg.*, **72**, 289.

NOGUCHI, Y. (1990). Blood vessel invasion in gastric carcinoma. *Surgery*, **107**, 140.

ROBERTS, S., JONASSON, O., LONG, L., MCGREW, E.,A., MCGRATH, R. & COLE, W.H. (1962). Relationship of cancer cells in the circulating blood to operation. *Cancer*, **15**, 232.

SUGANO, H., NAKAMURA, K. & KATO, Y. (1982). Pathological studies of human gastric cancer. *Acta. Pathol. Jpn.*, **32** (suppl 2), 329.

THÖRN, M., ADAMI, H.O., RINGBORG, U., BERGSTRÖM, R. & KRUSEMO, U.B. (1987). Long-term survival in malignant melanoma with special reference to age and sex as prognostic factors. *J. Natl Cancer Inst.*, **79**, 969.

TSUKUMA, H., MISHIMA, T. & OSHIMA, A. (1983). Prospective study of 'early' gastric cancer. *Int. J. Cancer*, **31**, 421.